# Functional comparison of SARS-CoV-2 with closely related pangolin and bat coronaviruses

**DOI:** 10.1038/s41421-021-00256-3

**Published:** 2021-04-06

**Authors:** Jianhui Nie, Qianqian Li, Li Zhang, Yang Cao, Yue Zhang, Tao Li, Jiajing Wu, Shuo Liu, Mengyi Zhang, Chenyan Zhao, Huan Liu, Lingling Nie, Haiyang Qin, Meng Wang, Qiong Lu, Xiaoyu Li, Junkai Liu, Haoyu Liang, Taijiao Jiang, Kai Duan, Xiaoming Yang, Yuelei Shen, Weijin Huang, Youchun Wang

**Affiliations:** 1 Division of HIV/AIDS and Sex-transmitted Virus Vaccines, Institute for Biological Product Control, National Institutes for Food and Drug Control (NIFDC) and WHO Collaborating Center for Standardization and Evaluation of Biologicals, No. 31 Huatuo Street, Daxing District, Beijing 102629, China; 2 Graduate School of Peking Union Medical College, No. 9 Dongdan Santiao, Dongcheng District, Beijing 100730, China; 3 Center of Growth, Metabolism and Aging, Key Laboratory of Bio-Resource and Eco-Environment of Ministry of Education, College of Life Sciences, Sichuan University, Chengdu, Sichuan 610065 China; 4 Center for Systems Medicine, Institute of Basic Medical Sciences, Chinese Academy of Medical Sciences & Peking Union Medical College, Beijing 100005, China; 5 Suzhou Institute of Systems Medicine, Suzhou, Jiangsu 215123 China; 6 China National Biotec Group Company Limited, Beijing 100029, China; 7 Beijing Biocytogen Co., Ltd., Beijing 101111, China

**Keywords:** Autoimmunity, Bioinformatics

## Abstract

The origin and intermediate host for severe acute respiratory syndrome coronavirus 2 (SARS-CoV-2) is yet to be determined. Coronaviruses found to be closely related to SARS-CoV-2 include RaTG13 derived from bat and two clusters (PCoV-GD and PCoV-GX) of coronaviruses identified in pangolin. Here, we studied the infectivity and antigenicity patterns of SARS-CoV-2 and the three related coronaviruses. Compared with the other three viruses, RaTG13 showed almost no infectivity to a variety of cell lines. The two pangolin coronaviruses and SARS-CoV-2 showed similar infectious activity. However, in SARS-CoV-2-susceptible cell lines, the pangolin coronaviruses presented even higher infectivity. The striking difference between the SARS-CoV-2 and pangolin coronaviruses is that the latter can infect porcine cells, which could be partially attributed to an amino acid difference at the position of 498 of the spike protein. The infection by SARS-CoV-2 was mainly mediated by Furin and TMPRSS2, while PCoV-GD and PCoV-GX mainly depend on Cathepsin L. Extensive cross-neutralization was found between SARS-CoV-2 and PCoV-GD. However, almost no cross-neutralization was observed between PCoV-GX and SARS-CoV-2 or PCoV-GD. More attention should be paid to pangolin coronaviruses and to investigate the possibility of these coronaviruses spreading across species to become zoonoses among pigs or humans.

## Introduction

Seven coronaviruses have been found to infect humans, including the four so-called common cold viruses HCoV-NL63, HCoV-229E, HCoV-OC43, and HKU1, as well as the three potentially lethal SARS-CoV, MERS-CoV, and SARS-CoV-2. SARS-CoV-2 caused the ongoing COVID-19 pandemic, which began in Dec. 2019, and has infected more than 110 million people with more than 2.4 million fatalities by Feb. 24, 2021 (https://www.who.int/emergencies/diseases/novel-coronavirus-2019/situation-reports/). Most of the coronaviruses that infect humans are believed to have originated from animals, among which SARS-CoV, MERS-CoV, HCoV-NL63, and HCoV-229E are believed to have originated from bats, while HCoV-OC43 and HKU1 are believed to have originated from rodents^1–3^. Moreover, intermediate hosts also play an important role in breaking the interspecies barrier and have been identified for some coronaviruses. The intermediate hosts of HCoV-229E and HCoV-OC43 are camelids and cows, while those of SARS-CoV and MERS-CoV are masked civets and dromedaries, respectively. SARS-CoV-2 is currently thought to have originated in bats, but its intermediate host is yet to be defined^4,5^.

Animal experiments showed that SARS-CoV-2 could infect monkeys^6^, hamsters^7^, ferrets^8^, cats^8^, tree shrews^9^, transgenic mice^10^, fruit bats^11^, dogs^12^ and minks^13^, but not pigs^8^ or poultry^11^. The closest relative of SARS-CoV-2 discovered to date is the bat coronavirus RaTG13 found in Yunnan, China, with a sequence similarity of 96.2%^14,15^. Recently, other SARS-CoV-2-related coronaviruses were identified from Malayan pangolins, which were genetically clustered into two clades, PCoV-GD and PCoV-GX^16,17^. PCoV-GD showed higher similarity to SARS-CoV-2 (91.2%) than PCoV-GX clade (85.4%)^18^. Although these pangolin-derived coronaviruses had lower overall homology with SARS-CoV-2 than RaTG13, their receptor-binding domain (RBD) regions were highly similar, and the six key RBD residues were completely identical for PCoV-GD and SARS-CoV-2^16^. Whether there is the possibility of cross-species transmission, and whether there is cross-protection by antibodies against these viruses, are all scientific questions requiring urgent attention.

## Results

### Significantly different infectivity of SARS-CoV-2 and coronaviruses derived from pangolins and bats

**Fig. 1:**
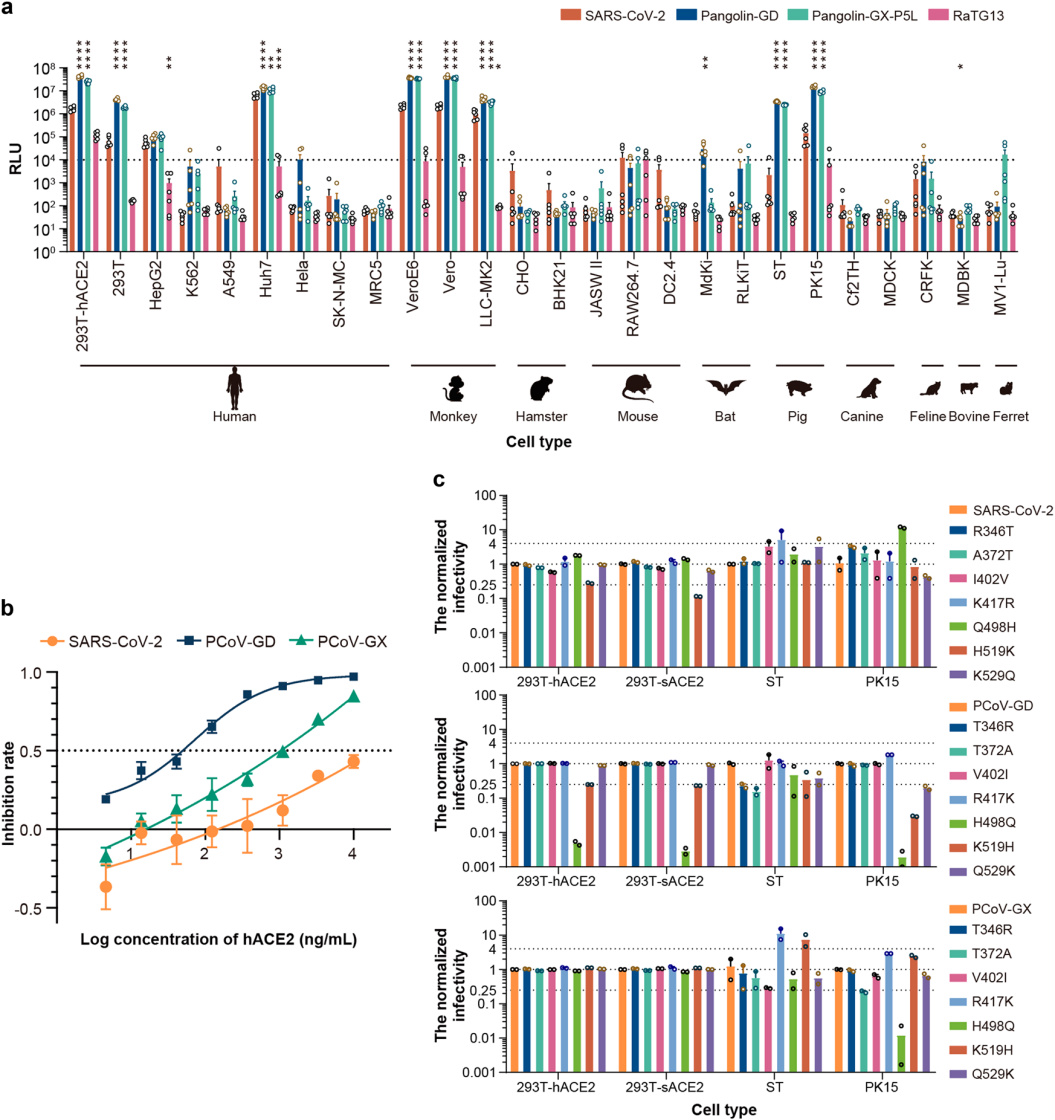
Infectivity analyses of the coronaviruses. **a** SARS-CoV-2, PCoV-GD, PCoV-GX, and RaTG13 pseudotyped viruses were used to infect a total of 26 cell lines. The infected cell lysates were analyzed for relative light units (RLU). All results were obtained from six independent experiments (mean ± SEM). In each experiment, the infection assay was performed in duplicate wells. The average value of the duplicate wells was presented in the plot as a circle. The one-way ANOVA and Dunnett’s multiple comparisons test were employed to study the differences of infectivity between SARS-CoV-2 and the other three coronaviruses. When the RLU for the tested cell reached 1% of that for 293T-hACE2 cell, it was deemed as a susceptible cell line. The dashed line indicates the 1% RLU value of the 293T-hACE2 cell. **b** The infections of SARS-CoV-2, PCoV-GD, PCoV-GX, and RaTG13 pseudotyped viruses to 293T-hACE2 could be inhibited by soluble hACE2. All results were obtained from six independent experiments (mean ± SEM). In each experiment, the infection assay was performed in duplicate wells. **c** The relative infectivity of the pseudotyped viruses with mutant S protein was presented as the ratio of RLU for mutants/RLU for original ones. All results were obtained from two independent experiments (mean ± SEM). In each experiment, the infection assay was performed in duplicate wells.

To study the infectivity of different SARS-CoV-2 closely related coronaviruses, we constructed spike (S) pseudotyped viruses^19^ for PCoV-GD, PCoV-GX, and RaTG13. Together with the previously constructed pseudotyped SARS-CoV-2^20^, the pseudotyped viruses were diluted to the same particle number based on quantitative analysis by RT-PCR^19,20^. Then, they were used to infect 26 cell lines derived from different species^21^ (Fig. 1a). Pseudotyped virus was defined as no-infectivity when its relative light unit (RLU) values decreased over 100-fold compared with the that of SARS-CoV-2 in 293T cells overexpressing human angiotensin-converting enzyme 2 (293T-hACE2 cells)^21^. We found that the RaTG13 S pseudotyped virus from bats was less infectious in all the tested cell types, and its infectivity spectrum was significantly different from that of SARS-CoV-2. By contrast, the infectivity spectrum of pangolin-derived PCoV-GD and PCoV-GX was much similar to SARS-CoV-2 in its susceptible cell lines. Compared with SARS-CoV-2, the infectivity of PCoV-GD and PCoV-GX became higher in SARS-CoV-2 susceptible cell lines, which were both about two-fold more infectious in Huh7 cells, 19- and 16-folds higher in Vero cells, and 4.5- and 3.2-folds higher in LLC-MK2 for PCoV-GD and PCoV-GX, respectively, than that of SARS-CoV-2. It is noteworthy that PCoV-GD and PCoV-GX also had significantly higher infectivity than SARS-CoV-2 in porcine cells (ST and PK15). The infectivity of PCoV-GD and PCoV-GX in ST cells was, respectively, higher by an average of 10,280- and 7387-folds, while the enhancements were 140 and 80 times in PK15 cells compared to SARS-CoV-2. Thus, the pangolin coronaviruses PCoV-GD and PCoV-GX showed a stronger ability to infect porcine cells, which have different infection profiles from SARS-CoV-2. Due to its poor infectivity for the RaTG13 pseudotyped virus in all the tested cell lines, it was not included in the following studies on infectivity and antigenicity.

### Infectivity of SARS-CoV-2 and pangolin coronaviruses depending on ACE2

To investigate whether infection by the pangolin-derived PCoV-GD and PCoV-GX coronaviruses depends on human ACE2 (hACE2) as is the case for SARS-CoV-2, 293T cells expressing hACE2 were infected with the three pseudotyped viruses, and the infection efficiency was in the order PCoV-GD > PCoV-GX > SARS-CoV-2. We further verified the dependence of the three viruses on hACE2. Soluble hACE2 was used to assess the inhibition of infection, and it was found that soluble hACE2 could effectively inhibit the infection of target cells by all three pseudotyped viruses (Fig. 1b). Soluble hACE2 had the highest inhibitory effect on PCoV-GD (EC_50_ = 5.1 ng/mL), followed by PCoV-GX (EC_50_ = 105.5 ng/mL), and SARS-CoV-2 (EC_50_ = 723.9 ng/mL). These results indicate that all three viruses can use the hACE2 receptor to infect cells, and pangolin-derived coronaviruses appear to have a higher affinity for hACE2 than SARS-CoV-2, for which it is the native receptor. Then, we compared the sequence difference between the RBDs of SARS-CoV-2, PCoV-GD, and PCoV-GX (Supplementary Fig. S1a). Compared with SARS-CoV-2, seven and 30 residue variations were observed in PCoV-GD, PCoV-GX, respectively. To investigate their impacts on inhibitory activity of soluble hACE2, we calculated the binding affinity between hACE2 and the RBDs of SARS-CoV-2, PCoV-GD, and PCoV-GX^22^. The results showed that PCoV-GD bound with hACE2 obviously stronger than SARS-CoV-2 and PCoV-GX (Supplementary Fig. S1b), which could explain why soluble hACE2 showed higher inhibitory activity against PCoV-GD than against PCoV-GX and SARS-CoV-2.

### The key site differences influencing the infectivity

To investigate why the two pangolin coronaviruses can infect porcine cells, we compared the RBD amino acid sequences of SARS-CoV-2, PCoV-GD, and PCoV-GX. We found that SARS-CoV-2 had the highest sequence similarity with PCoV-GD, and only seven amino acids were different in RBD (Supplementary Fig. S1a). The seven corresponding amino acids of SARS-CoV-2 were then mutated into the same amino acids as those in PCoV-GD. Conversely, the seven amino acids corresponding to PCoV-GD and PCoV-GX were mutated into the same amino acids as those of SARS-CoV-2. The S-protein-expressing plasmids with RBD mutations were then packaged into 21 mutant pseudotyped viruses. These 21 pseudotyped viruses were then used to infect cells overexpressing hACE2 and swine ACE2 (sACE2), as well as ST and PK15 cells (Fig. 1c). We found that when the amino acid at position 498 of SARS-CoV-2 was changed from glutamine to histidine, the infectivity in 293T-hACE2 was slightly increased (~2-fold higher). Conversely, when the corresponding amino acid in PCoV-GD was mutated from histidine to glutamine, its infectivity in 293T-hACE2 was significantly reduced (~206-fold lower). In PCoV-GX, when the corresponding amino acid was mutated from histidine to glutamine, almost no infectivity change was observed in 293T-hACE2. When we tested these mutant pseudotyped viruses in porcine-related cells, even significant changes were found. Q498H mutation could drastically enhance the infectivity of SARS-CoV-2 in porcine cells, especially in porcine PK15 cells, where it was increased 12 times. Conversely, when the corresponding amino acid in PCoV-GD was mutated from histidine to glutamine, the infectivity of PCoV-GD with H498Q mutation in 293T-sACE2, ST, and PK15 cells was significantly reduced and was respectively 206, 350, and 590 times lower than that of the original strain. H498Q mutation in PCoV-GX also reduced its infectivity in porcine cell lines. PK15 cells showed the greatest difference, with a 162-fold reduction compared to the wild type.

### The role of proteases in SARS-CoV-2, PCoV-GD, and PCoV-GX infection

**Fig. 2:**
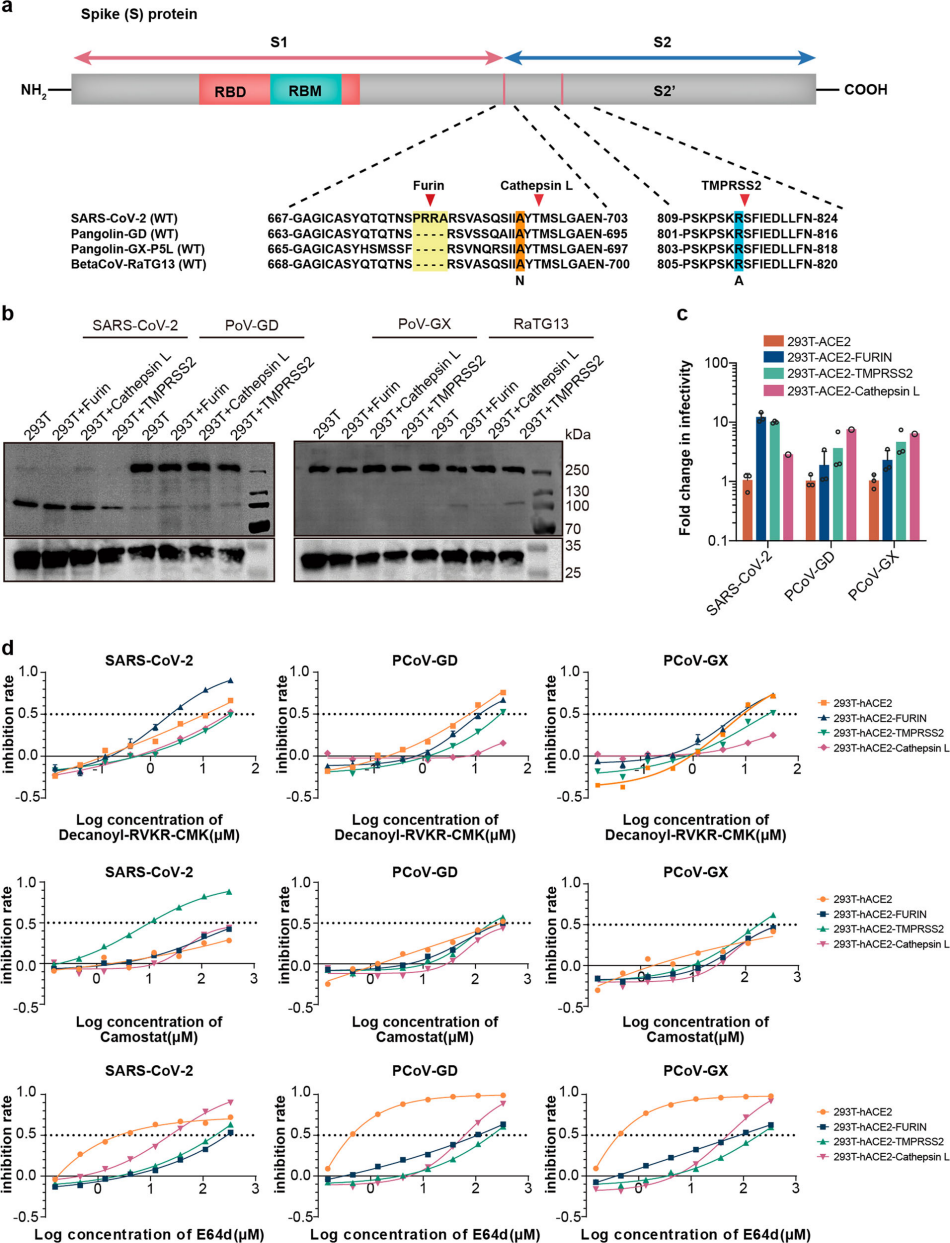
Effects of protease cleavage on viral infectivity. **a** Amino acid alignment of the protease cleavage site for SARS-CoV-2, PCoV-GD, PCoV-GX, and RaTG13. The recognition sequences were embraced in boxes and cleavage sites indicated with triangles. **b** Pseudoviruses produced in cells overexpressing different proteases. Most of SARS-CoV-2 S were cleaved into S1 and S2. The ratio of the cleaved form increased slightly in Furin and TMPRSS2. The other three pseudoviruses present the S on their surface mostly in intact forms. **c** Relative infectivity of the pseudoviruses in hACE2 cells and hACE2 cells with various overexpressed proteases. **d** The entry of SARS-CoV-2, PCoV-GD, and PCoV-GX into cell lines expressing different proteases was blocked with three protease inhibitors, Decanoyl-RvKR-CMK for Furin, Camostat for TMPRSS2, and E64D for Cathepsin L inhibitor. All results were obtained from three independent experiments (mean ± SEM). In each experiment, the infection assay was performed in duplicate wells.

In addition to receptors on the target cell surface^14^, cellular enzymes may also affect viral infectivity^23–27^. To investigate the role of proteases in SARS-CoV-2, PCoV-GD, and PCoV-GX infection, we compared the sequences of the S proteins of the three viruses and found that all three have recognition sites for cleavage by TMPRSS2 and Cathepsin L, but only SARS-CoV-2 has a Furin cleavage site (Fig. 2a). First, we investigated the role of the three proteases in the packaging process of different viruses. To produce pseudotyped viruses, cells overexpressing Furin, TMPRSS2, and Cathepsin L were transfected with SARS-CoV-2, PCoV-GD, and PCoV-GX spike expressing plasmids together with infection of VSV G pseudotyped virus^19^, which showed that SARS-CoV-2 containing a Furin recognition site expressed both enzymatically cleaved and uncleaved proteins in 293T cells, mainly in the cleaved form. However, in cells with Furin or TMPRSS2 overexpression, the proportion of the processed form increased further, while proteins expressed in the Cathepsin L-overexpressing cells were not processed. The S proteins of PCoV-GD and PCoV-GX coronaviruses were also not processed, both in the cells without protease overexpression or those overexpressing Furin, TMPRSS2, and Cathepsin L. Different from SARS-CoV-2 with a cleaved form of S1 and S2 on the viral surface, both PCoV-GD and PCoV-GX present intact S protein on their surface, indicating a lack of protease processing during the virus packaging process (Fig. 2b).

Next, we investigated the role of cellular proteases in infectivity. SARS-CoV-2, PCoV-GD, and PCoV-GX S pseudotyped viruses were used to infect cells expressing hACE2 and different proteases respectively (Fig. 2c). We found that the infectivity of the three viruses increased to different degrees in the protease-overexpressing cells. Among them, SARS-CoV-2 infectivity was most significantly increased in cells overexpressing Furin and TMPRSS2, in which it was 11 times and nine times more infectious than in cells overexpressing hACE2 alone. Moreover, SARS-CoV-2 infectivity was nearly two times higher in Cathepsin L-overexpressing cells. The infectivity of the two pangolin coronaviruses showed similar trends in the three protease-overexpressing cell lines. The infectivity of PCoV-GD and PCoV-GX were both two times higher in Furin-overexpressed cells, three and four times higher in TMPRSS2-overexpressing cells, as well as eight and six times higher in Cathepsin L-overexpressing cells, respectively. These results indicate that protease activation is required when the three coronaviruses infect cells, but infection by SARS-CoV-2 mainly relies on Furin and TMPRSS2, while PCoV-GD and PCoV-GX mainly depend on Cathepsin L.

To further verify the role of proteases in viral infection, three protease inhibitors, Decanoyl-RvKR-CMK (Furin inhibitor), Camostat (TMPRSS2 inhibitor), and E64D (Cathepsin L inhibitor), were used to inhibit viral infection (Fig. 2d). The Furin inhibitor inhibited the infection of 293T-hACE2 cells overexpressing Furin by pseudotyped SARS-CoV-2. Similarly, the TMPRSS2 inhibitor also inhibited infection of cells overexpressing TMPRSS2. By contrast, the Cathepsin L inhibitor had a weak effect on the infectivity of SARS-CoV-2 infectivity. Similarly, the Furin and TMPRSS2 inhibitors showed no obvious inhibition on the two pangolin coronaviruses, while the Cathepsin L inhibitor had an obvious inhibitory effect on the infection of 293T-hACE2 cells with PCoV-GD and PCoV-GX. These results indicate that all three proteases play a role in SARS-CoV-2 infection, while Cathepsin L is the main protease mediating infection by PCoV-GD and PCoV-GX.

### Differential cross-neutralization activity between SARS-CoV-2 and pangolin coronaviruses

**Fig. 3:**
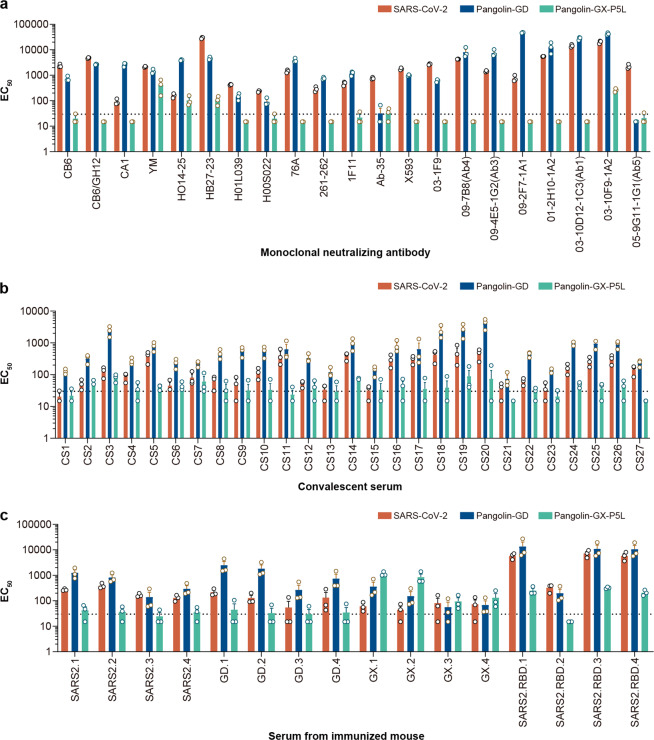
Antigenicity analyses of SARS-CoV-2, PCoV-GD, and PCoV-GX. **a** Nineteen SARS-CoV-2 monoclonal neutralizing antibodies were used to test against SARS-CoV-2, PCoV-GD, and PCoV-GX. **b** Twenty-seven convalescent serum samples from SARS-CoV-2-infected individuals were employed to investigate the cross-reactivity between the three pseudotyped viruses. **c** S-protein-expressing plasmids for SARS-CoV-2, PCoV-GD, and PCoV-GX were used to immunize animals respectively. SARS2, GD, and GX represent SARS-CoV-2, PCoV-GD, and PCoV-GX, respectively. The numbers showed the animal code; 1 and 2 indicate two serum pools (five mice for each) from immunized mice, 3 and 4 were designated to two individual serum samples from guinea pigs. Extensive cross-neutralization was observed between SARS-CoV-2 and PCoV-GD, but almost no cross-reactivity between PCoV-GX and them.

Besides the infectivity of these coronaviruses, the characteristics of their antigenicity is another important question to be addressed. To study the differences in the antigenicity of SARS-CoV-2, PCoV-GD, and PCoV-GX, we first selected 19 neutralizing monoclonal antibodies against SARS-CoV-2 S protein and tested their effects on the three pseudotyped viruses (Fig. 3a). We found that 18 of these monoclonal antibodies were effective in neutralizing the PCoV-GD pseudotyped virus. Remarkably, 11 of them were more effective in neutralizing both PCoV-GD than SARS-CoV-2. However, these monoclonal antibodies had a poor neutralizing effect on the PCoV-GX pseudotyped virus. Only two had a weak neutralizing effect on PCoV-GX pseudotyped virus, and the neutralizing effect was significantly lower than against SARS-CoV-2 or PCoV-GD. This indicates that neutralizing monoclonal antibodies against SARS-CoV-2 S protein can cross-neutralize PCoV-GD with high efficiency, while their cross-neutralization effect on PCoV-GX is weak.

Next, the convalescent sera of recovered COVID-19 patients were used to further verify the scope of cross-neutralization between the three viruses (Fig. 3b). We selected convalescent sera from 27 recovered COVID-19 patients to conduct neutralization tests on the three pseudotyped viruses and found that similar to the monoclonal antibody neutralization results, 26 of the 27 sera were able to neutralize the SARS-CoV-2 pseudotyped virus and all 27 effectively neutralized the PCoV-GD pseudotyped virus with higher effectiveness than for SARS-CoV-2. However, the cross-neutralization effect of these convalescent sera on PCoV-GX was weak, and only nine could neutralize PCoV-GX pseudotyped virus, whereby the neutralizing titer was significantly lower than for SARS-CoV-2 or PCoV-GD.

To further verify the cross-reactivity of neutralizing antibodies against the three S proteins, we immunized mice and guinea pigs with three S-protein-expressing plasmids and collected the sera for cross-neutralization assay with the three pseudotyped viruses (Fig. 3c). The serum neutralizing activity of animals immunized with the SARS-CoV-2 S protein expression plasmid was in the order PCoV-GD > SARS-CoV-2 > PCoV-GX. The serum of animals immunized with the PCoV-GD S protein expression plasmid was similar to that of SARS-CoV-2, and the order of neutralizing activity was PCoV-GD > SARS-CoV-2 > PCoV-GX. Compared with the sera immunized with the SARS-CoV-2 S-protein-expressing plasmid, the PCoV-GD S protein induced higher neutralization activity against its pseudotyped virus. However, the sera of animals immunized with SARS-CoV-2 and PCoV-GD S-protein-expressing plasmids had almost no neutralizing effect on PCoV-GX. The sera of mice immunized with the PCoV-GX S protein expression plasmid had strong neutralizing activity against its pseudotyped virus but had a weak neutralizing activity against PCoV-GD and SARS-CoV-2. These results indicated that there is strong immunological cross-reactivity between SARS-CoV-2 and PCoV-GD, and the pseudotyped PCoV-GD was more easily neutralized. The cross-neutralization reaction of PCoV-GX with SARS-CoV-2 and PCoV-GD was weak, and the ability of PCoV-GX S protein to induce neutralizing antibodies was also weak.

## Discussion

The identification of intermediate hosts for potential pandemic pathogens is critical for prevention and control. However, there is still controversy over the intermediate host of SARS-CoV-2. Its closest relative found to date appears to be RaTG13, a bat coronavirus found in the Pu’er area of Yunnan province, with 96.2% nucleotide sequence similarity over the whole genome^14^. In addition to RaTG13, the coronaviruses derived from pangolin have been found to exhibit strong similarity to SATS-CoV-2, especially in the RBD^28^. There are two main types of pangolin coronaviruses, which are related to the geographical location of their discovery. The coronavirus PCoV-GD (91.2% sequence similarity to SARS-CoV-2), isolated from pangolins imported from Guangdong^16^, is more closely related to SARS-CoV-2, while the coronavirus PCoV-GX (85.4% sequence similarity), isolated from pangolins imported from Guangxi^17^, is more distant. Moreover, the protein sequence similarity of pangolin coronaviruses with SARS-CoV-2 is even higher, with PCoV-GD and SARS-CoV-2 sharing 94.1% of their encoded amino acids. We also found that among viruses closely related to SARS-CoV-2, the sequence similarity of the RBD from bat coronaviruses to the RBD of SARS-CoV-2 is lower than that of S protein. Conversely, the sequence similarity of the RBD from pangolin coronaviruses to that from SARS-CoV-2 is higher than that of the S protein. In particular, the RBD region of PCoV-GD has 96.8% amino acid sequence similarity with the RBD of SARS-CoV-2, with only seven different amino acids. Moreover, only one amino acid at the site that directly binds to ACE2 differs between SARS-CoV-2 and PCoV-GD. Hence, the 498th amino acid of SARS-CoV-2 is glutamine, while the corresponding residue of PCoV-GD is histidine, which is also the only difference between the two viruses in the whole RBM. However, the H498Q mutation could significantly reduce the infectivity of PCoV-GD but has almost no effect on infectivity of PCoV-GX in human cells. To investigate the possible reasons for this phenomenon, we performed the computational structural modeling of ACE2-RBD complexes for PCoV-GD, PCoV-GX, and their mutants of H498Q (Supplementary Fig. S2). It was observed that the residue H498 of PCoV-GD and the residue D38 of ACE2 contacted with each other. The oxygen–nitrogen atom distance was ~3.7 Å which is able to form a salt bridge (the distance required is less than 4 Å) (Supplementary Fig. S2a). On the contrary, the Q498 showed larger distance (~4.6 Å) with the D38 of ACE2, which could not form a salt bridge and was out of the range of hydrogen bond (Supplementary Fig. S2b). Hence, the H498Q strongly reduced the binding affinity with ACE2. In PCoV-GX, the residue H498 may pack in a different side-chain orientation to form a hydrogen bond with its neighbor residue T445 (Supplementary Fig. S2c). It was different from the V445 of PCoV-GD which cannot attract H498 to form a hydrogen bond. Similarly, the mutant H498Q of PCoV-GX formed a hydrogen bond with T445 (Supplementary Fig. S2d). In conclusion, those models suggest the mechanism that the H498Q in PCoV-GD significantly reduced the infectivity with no effect for PCoV-GX.

SARS-CoV-2 has a different infection profile from the pangolin coronaviruses PCoV-GD and PCoV-GX, which showed a stronger ability to infect porcine cells. The mutation of the 498th amino acid of SARS-CoV-2 from glutamine to histidine significantly enhanced the infectivity of the SARS-CoV-2 pseudotyped virus in porcine cells. Correspondingly, the infectivity of PCoV-GD and PCoV-GX pseudotyped viruses in porcine cells was significantly reduced when the 498th amino acid was mutated from histidine to glutamine. In the S protein of SARS-CoV-2, there are three hydrogen bonds and 20 van der Waals’ force interactions between the 498th amino acid, which is located at the core of the binding domain, and hACE2^29^. Therefore, it may be the key site in the S protein affecting the infection of porcine cells. The infectivity of PCoV-GD and PCoV-GX pseudotyped viruses in porcine cells suggested that the virus might infect pigs through ACE2, thus causing epidemics among pigs. Moreover, PCoV-GD and PCoV-GX can also infect human cells, which may lead to cross-species transmission of the virus between humans, pigs, and pangolins, possibly leading to the emergence of a new zoonotic disease. Although SARS-CoV-2 cannot infect porcine cells, the Q498H mutation significantly improved the infectivity of SARS-CoV-2 in porcine cells. Two SARS-CoV-2 sequences carrying the Q498H mutation in spike have been deposited in GISAID database up to Feb. 24, 2021. Additionally, a mutational scanning study demonstrated that this mutation could enhance the affinity of S to hACE2^30^. Due to its potential to enhance infectivity, variants carrying Q498H mutation should be closely monitored and attention should be paid to the cross-species transmission of the mutant virus.

Although the available data do not support the hypothesis that SARS-CoV-2 is derived from pangolins, or that pangolins are the intermediate hosts linking bats with humans^31^, PCoV-GD and PCoV-GX can infect both human and non-human cells, and their infectivity is even higher than that of SARS-CoV-2. These findings suggest that pangolin coronaviruses may also have the potential for cross-species transmission to humans. Also, we found extensive cross-neutralization between SARS-CoV-2 and PCoV-GD in antigenicity studies. Neutralizing monoclonal antibodies raised against SARS-CoV-2, vaccine-induced animal sera, as well as convalescent sera from recovered COVID-19 patients were able to neutralize pseudotyped PCoV-GD, and the neutralization activity was even higher than against pseudotyped SARS-CoV-2. The animal antisera induced by DNA expressing PCoV-GD S protein were also able to neutralize pseudotyped SARS-CoV-2. Therefore, if the current SARS-CoV-2 vaccine candidates and neutralizing antibodies prove effective against SARS-CoV-2, they will also be able to prevent or treat PCoV-GD infection to a large extent. The second pangolin coronavirus investigated in this study, PCoV-GX, has a similar ability to infect human cells to that of PCoV-GD, but corresponding antibodies had a weak cross-neutralization effect on SARS-CoV-2. The existing SARS-CoV-2 vaccine candidates and neutralizing antibodies are therefore likely to be ineffective against PCoV-GX. We should therefore strengthen the screening and monitoring of coronaviruses in pangolins and other species, as well as to conduct more research on the mechanisms of cross-species transmission of coronaviruses. These studies can lay a foundation for prevention and control, and enable the development of a technical reserve for possible cross-species transmission events, and in the worst-case scenario, new pandemics.

In conclusion, the bat coronavirus RaTG13 is highly similar to SARS-CoV-2 at the whole-genome level, but its ability to infect the cells of other mammals is significantly different from SARS-CoV-2, which may be due to the large difference in the RBD region. The infectivity of the two pangolin coronaviruses investigated in this study was closer to that of SARS-CoV-2, which can, in turn, be explained by their more similar RBD. Notably, their infectivity in porcine cells was significantly higher than that of SARS-CoV-2. The antigenicity of PCoV-GD and SARS-CoV-2 was close, and there was strong cross-neutralization between them. By contrast, there was almost no cross-reactivity between PCoV-GX and either SARS-CoV-2 or the other pangolin coronavirus investigated here, indicating that it represents a distinct serotype. In the future, we should closely monitor the spread of different coronaviruses similar to SARS-CoV-2, explore the origins and intermediate hosts of SARS-CoV-2, investigate the possibility of other coronaviruses spreading across species to become zoonosis, and study corresponding prevention and control strategies, so as to avoid new coronaviruses causing new pandemics.

## Materials and methods

### Plasmids

Five spike protein expression plasmids, three protease expression plasmids, two ACE2 receptor expression plasmids, and 21 spike protein point mutation expression plasmids were constructed, including: SARS-CoV-2 spike (GenBank: MN908947), PCoV-GD spike (GISAID: EPI_ISL_410721), PCoV-GX spike (GISAID: EPI_ISL_410540), RaTG13 spike (GISAID: EPI_ISL_402131), Furin (GenBank: NM_002569.2), TMPRSS2 (GenBank: NM_005656.3), Cathepsin L (GenBank: NM_145918.2), hACE2 (GenBank: BAB40370.1), sACE2 (GenBank: NP_001116542.1). These protein expression plasmids were synthesized by General Biol. Co., Ltd. All the sequences were codon-optimized for mammalian cells and inserted into the eukaryotic expression vector pcDNA3.1 between the *Bam*HI and *Xho*I restriction sites to obtain the plasmids pcDNA3.1-SARS-CoV-2-spike, pcDNA3.1-PCoV-GD-spike, pcDNA3.1-PCoV-GX-spike, pcDNA3.1-RaTG3-spike, pcDNA3.1-Furin, pcDNA3.1-TMPRSS2, pcDNA3.1-Cathepsin L, pcDNA3.1-hACE2, and pcDNA3.1-sACE2, respectively. The point mutation plasmids were constructed based on the spike protein expression plasmids using the primers listed in Supplementary Table S1.

### Cells

Most cell lines were cultured in Dulbecco’s modified Eagle medium (DMEM, high glucose; HyClone). They included 293T (American Type Culture Collection [ATCC], CRL-3216), Huh-7 (Japanese Collection of Research Bioresources [JCRB], 0403), HepG2 (ATCC, HB-8065), HeLa (ATCC, CCL-2), MRC-5 (ATCC, CCL-171), A549 (ATCC, CCL-185), Hep2 (ATCC, CCL-23), Vero (ATCC, CCL-81), Vero E6 (ATCC, CRL-1586), LLC-MK2 (ATCC, CCL-7), PK15 (ATCC, CCL-33), ST (ATCC, CRL-1746), BHK21 (ATCC, CCL-10), BHK-T7 (stable BHK-21 cell line constitutively expressing bacteriophage T7 RNA polymerase), CHO (ATCC, CCL-61), NIH/3T3 (ATCC, CRL-1658), RAW264.7 (ATCC, TIB-71), MDCK (ATCC, CCL-34), Cf2TH (ATCC, CRL-1430), CRFK (ATCC, CCL-94), MDBK (ATCC, CCL-22), and MV1-Lu (ATCC, CCL-64) cells. DC2.4 (Millipore, SCC142), K562 (ATCC, CCL-243), and MdKi^32^ (Laboratory of Dr. Zhengli Shi, Wuhan Institute of Virology, Chinese Academy of Sciences) cells were incubated in modified RPMI medium (HyClone). RlKiT^32^ cells (Laboratory of Dr. Zhengli Shi) were cultured in DMEM/F-12, GlutaMAX (GIBCO). All the cells were cultured in media supplemented with 100 U/mL of penicillin–streptomycin solution (GIBCO), 20 mM *N*-2-hydroxyethylpiperazine-*N*-2-ethane sulfonic acid (HEPES, GIBCO), and 10% fetal bovine serum (FBS, Pansera ES, PAN-Biotech) at 37 °C in a humidified atmosphere comprising 5% CO_2_. Where appropriate, 0.25% trypsin-EDTA (GIBCO) was used to detach cells for subculture every 2–3 days.

To construct Furin-, TMPRSS2-, Cathepsin L-, hACE2-, and sACE2-overexpressing cell lines, 293T cells were transfected with 30 μg of pcDNA3.1-Furin, pcDNA3.1-TMPRSS2, pcDNA3.1-Cathepsin L, pcDNA3.1-hACE2, or pcDNA3.1-sACE2 using Lipofectamine 2000 (Invitrogen) transfection reagent to obtain transient cell lines 293T-Furin, 293T-TMPRSS2, 293T-Cathepsin L, 293T-hACE2, and 293T-sACE2, respectively. The cell culture medium was the same as that used for 293T cells. After transfection for 24 h, the cells were used for subsequent experiments.

To construct cell lines co-expressing hACE2 protein and Furin, TMPRSS2 or Cathepsin L, 293T cells were transfected with 15 μg of pcDNA3.1-hACE2 and 15 μg of pcDNA3.1-Furin, 15 μg of pcDNA3.1-TMPRSS2 or 15 μg of pcDNA3.1-Cathepsin L to obtain 293T-hACE2-Furin, 293T-hACE2-TMPRSS2, and 293T-hACE2-Cathepsin L transient cell lines, respectively. The cell culture medium was the same as that used for 293T cells, and the cells were used for further experiments following 24 h after transfection.

### Monoclonal antibodies

A total of 19 neutralizing monoclonal antibodies against SARS-CoV-2 S protein were tested. CB6 and CA1 were provided by Jinghua Yan, Institute of Microbiology, Chinese Academy of Sciences^33^; H014, HB27, H01L039, and H00S022 were provided by Liangzhi Xie, Sino Biological Company; 76A, 261-262, 1F11, and Ab-35 were provided by Linqi Zhang, Tsinghua University^34^; X593 was provided by Sunney Xie, Peking University; s03-1F9, 09-7B8, 09-4E5-1G2, 09-2F7-1A1, 01-2H10-1A2, 03-10D12-1C3, 03-10F9-1A2, and 05-9G11-1G1 were provided by Beijing Biocytogen Co., Ltd.

### Convalescent sera

The sera of 27 convalescent patients from Wuhan (CS1-20) and Shandong (CS21-27) were provided by China National Biotec Group Company and China Biologic Products Holdings, respectively. This study (No. 2020026) was approved by the Ethics Committee of the Beijing Center for Disease Prevention and Control.

### Animal immunization

Mouse sera were obtained by immunizing ten SPF BALB/c mice with pcDNA3.1-SARS-CoV-2-spike, pcDNA3.1-PCoV-GD-spike, and pcDNA3.1-PCoV-GX-spike plasmids. The mice were immunized via electroporation, once every 2 weeks, 50 μg each time, and a total of three times. Blood samples were collected 7 days after the third immunization after anesthetized. The sera of five mice were mixed and labeled as SARS2.1, SARS2.2, GD.1, GD.2, GX.1, and GX.2, respectively. Guinea pig sera were obtained by immunizing four SPF Hartley guinea pigs with pcDNA3.1-SARS-CoV-2-spike, pcDNA3.1-PCoV-GD-spike, and pcDNA3.1-PCoV-GX-spike plasmids. The guinea pigs were immunized via electrical stimulation once every 2 weeks, 200 μg each time, and a total of three times. Blood samples were collected 7 days post the third immunization after anesthetized. The sera of two guinea pigs were mixed and labeled as SARS2.3, SARS2.4, GD.3, GD.4, GX.3, and GX.4, respectively.

Mouse sera (SARS2.RBD.1-4) were obtained by immunizing SPF BALB/c mice with SARS-CoV-2 spike RBD protein. The recombinant RBD protein was mixed with aluminum adjuvant and injected subcutaneously. The mice were immunized once every other week for three times. Blood samples were collected 7 days after the third immunization.

The protocol 2020 (B) 01 was approved by the Ethical Review Committee for Animal Welfare of The National Institutes for Food and Drug Control.

### Preparation of pseudotyped viruses

Pseudotyped viruses of SARS-CoV-2, PCoV-GD, PCoV-GX, and RaTG13 S and point mutants were constructed according to our previous study. The 293T cells or Furin-, TMPRSS2-, and Cathepsin L-overexpressing cells were digested and adjusted to concentrations of 5 × 10^5^–7 × 10^5^ cells/mL the day before transfection. Then, cells in 15 mL of medium were transferred to a T75 cell culture flask and incubated overnight at 37 °C in an incubator with 5% CO_2_. When the cells reached 70%–90% of confluence, the medium was discarded and 15 mL of G*ΔG-VSV virus (VSV G pseudotyped virus, Kerafast) at a concentration of 7 × 10^4^ TCID_50_/mL was used for infection. At the same time, the cells were transfected with 30 μg of S protein expression plasmid according to the user manual and then cultured in an incubator with 5% CO_2_ at 37 °C. The cell supernatant was discarded after 6–8 h and the cells were gently rinsed twice with PBS + 1% FBS. Next, 15 mL of fresh complete DMEM was added to the T75 cell culture flask, and after 24 h of culture in an incubator at 37 °C and 5% CO_2_, the supernatant containing pseudotyped virus-containing culture supernatant was harvested, filtered, aliquoted, and frozen at −70 °C for further use.

### Pseudotyped virus infection test

RNA of SARS-CoV-2, PCoV-GD, PCoV-GX, RaTG13 S pseudotyped viruses, and point-mutated pseudotyped viruses were extracted, and DNA was obtained by reverse transcription. After quantitative analysis by RT-PCR^19,20^, the pseudotyped viruses were diluted to the same particle number, and 100 μL was taken and added into a 96-well cell culture plate. A total of 26 different cell lines, including hACE2- and sACE2-overexpressing cells were digested by trypsin and 2 × 10^4^/100 μL cells were added to each well of the 96-well plate. Then, after incubation in a 37 °C incubator with 5% CO_2_ for 24 h, chemiluminescence detection was conducted^19^. To each well, 100 μL of luciferase substrate (PerkinElmer) was added, incubated at room temperature for 2 min, and then transferred to the detection whiteboard and measured using a luminometer (PerkinElmer). Each group contained six replicates.

### Pseudotyped virus infection inhibition test

The reduction of luciferase gene expression was detected to evaluate the infection inhibition effect of hACE2 protein (expressed in HEK293 cells, Sino Biological), protease inhibitor Decanoyl-RVKR-CMK (Furin inhibitor, R&D Systems), protease inhibitor Camostat (TMPRSS2 inhibitor, Tocris Bioscience), protease inhibitor E64D (Cathepsin L inhibitor, APExBIO), monoclonal antibody, and sera. As described previously^19,20^, the tested samples were diluted (starting with 30 times dilution and three times gradient, a total of eight gradients), after which the virus solution was added. On each 96-well plate, eight virus control wells and eight cell control wells were arranged. In the virus control wells, no test sample but only virus solution was added; in the cell control wells, no virus solution but only a complete culture medium was added. The 96-well plates were incubated at 37 °C for 1 h, after which trypsinized Huh7 cells (2 × 10^4^/100 μL) were added to each well. After incubation for 24 h in an atmosphere comprising 5% CO_2_ at 37 °C, luminescence was measured as described above. The EC_50_ value of each sample was calculated using the Reed–Muench method.

### Western blot analysis

An aliquot comprising 7 mL of pseudotyped virus solution was added to a 2 mL 25% sucrose buffer and centrifuged at 100,000 × *g* for 3 h. The purified virus particles were then re-suspended in 100 μL of PBS buffer, and 20 μL of 6× SDS sample buffer was added to 100 μL of the re-suspended pseudotyped virus, mixed well, and heated in a 100 °C metal bath for 5 min. Then, a 15 μL sample was used for SDS-PAGE and western blot analysis. The protein ladder (Thermo Fisher Scientific, Cat: 26619) was loaded as a molecular weight marker.

A 500-fold dilution of SARS-CoV-2 (2019-nCoV) spike antibody was used as the primary antibody. The polyclonal antibody was harvested 7 days after thrice immunization of SPF balb/c mice with purified S2 protein. A 1:10,000 dilution of HRP-conjugated goat anti-mouse IgG (CW Biotech) was used as the secondary antibody. The VSV M protein was blocked as an internal reference using VSV-M antibody (23H12, Cat: EB0011, kerafast). Immobilon Western chemiluminescent HRP substrate (Millipore) was used to develop the immune-reactive bands.

### Structure modeling

The complex crystal structure (PDB code: 6M0J)^35^ of SARS-CoV-2 spike RBD bound with ACE2 was obtained from Protein Data Base to be the modeling template. For a given protein sequence of spike protein or ACE2, we align the sequence to the template and introduction residue substitutions in 3D structure without perturbing the protein backbone conformation using a well-established clash-detection guided iterative search algorithm^36^. Next, we refined the structures using molecular dynamics method NAMD^37^ for 5000 steps of energy minimization. The binding affinities between the RBDs and ACE2 were computed with an empirical binding affinity scoring function^22^. The figures were drawn with PyMol (Schrodinger, LLC. 2010).

### Statistical analysis

The one-way ANOVA and Dunnett’s multiple comparisons test were used to study the differences of infectivity between SARS-CoV-2 and the other three coronaviruses. GraphPad Prism 8 was used for plotting and statistical analysis.

## Supplementary information

Supplemental Information
