# Supplementary material for: Functional comparison of SARS-CoV-2 with closely related pangolin and bat coronaviruses
Source: Cell Discov. 2021 Apr 6;7:21. doi: 10.1038/s41421-021-00256-3 (PMC8022302; doi:10.1038/s41421-021-00256-3)
Supplement: Supplementary file 1 — Supplemental Information [file 41421_2021_256_MOESM1_ESM.pdf]

**List of supplementary materials:**

Table S1

Figure S1-S2

Supplementary Table S1. Primers used for site-directed mutagenesis

| **Name** | **Sequence (5‘-3‘)** |
|---|---|
| SC2-R346T-F | CGAGGTGTTCAATGCCACCACTTTCGCCAGCGTGTA |
| SC2-A372T-F | GCTGTACAATAGCACCAGCTTCAGCACCTTCAAATGTTATGG |
| SC2-I402V-F | CGCCGACAGCTTCGTGGTTAGAGGCGACGAGGTGAG |
| SC2-K417R-F | CCAGGGCAGACCGGCCGTATCGCCGACTACAATTACAA |
| SC2-Q498H-F | GCAGAGCTACGGCTTCCATCCTACCAATGGCGTGGGCTA |
| SC2-H519N-F | GCTGAGCTTCGAGCTGCTGAATGCTCCCGCTACCGTGT |
| SC2-K529Q-F | GTGTGCGGCCCTAAGCAAAGCACCAATCTGGTGAAGA |
| GD-T342R-F | GAGGTGTTTAATGCCACACGTTTCGCCAGCGTGTACGCCTGG |
| GD-T368A-F | CTCCGTGCTGTACAATTCCGCCTCCTTCAGCACCTTTA |
| GD-V398I-F | CGCCGACTCCTTCGTGATTAGGGGCGACGAGGTGAGAC |
| GD-R413K-F | GCCCCTGGCCAGACAGGCAAAATCGCCGACTACAACTA |
| GD-H494Q-F | GCTACGGCTTTCAACCCACAAACGGCGTGGGCTACC |
| GD-N515H-F | TGAGCTTTGAGCTGCTGCATGCCCCCGCCACCGTGT |
| GD-Q525K-F | ACCGTGTGTGGCCCTAAGCAAACCACCAACCTGGTGAAG |
| GX-K344R-F | GTTTAATGCCAGCCGTTTTGCCTCCGTGTACGCCTGGA |
| GX-T370A-F | CTCCGTGCTGTACAATTCCGCATCCTTTAGCACATTCA |
| GX-V400I-F | GATAGCTTTGTGATTAAGGGCGACGAGGTGAGGCAGAT |
| GX-V415K-F | GCCAGACAGGCAAAATCGCCGACTACAACTACAAGC |
| GX-H496Q-F | GGAGAGGTACGGCTTTCAACCCACCACCGGCGTGAATTA |
| GX-N517H-F | TGTCCTTTGAGCTGCTGCATGGCCCCGCCACCGTGT |
| GX-L527K-F | TGTGTGGCCCAAAGAAAAGCACCACCCTGGTGAAGGATA |

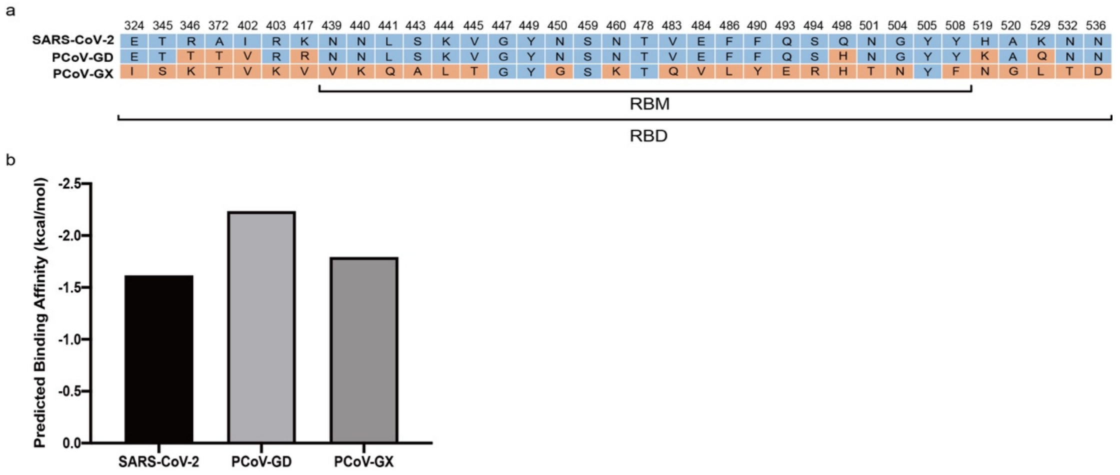


**Supplementary Fig. S1 Comparison of RBDs for SARS-CoV-2, PCoV-GD, and PCoV-GX. a** Sequence comparison of RBDs for SARS-CoV-2, PCoV-GD, and PCoV-GX. Only the different amino acids of RBDs were presented. **b** Calculated binding affinity of RBDs for SARS-CoV-2, PCoV-GD, and PCoV-GX to human ACE2.

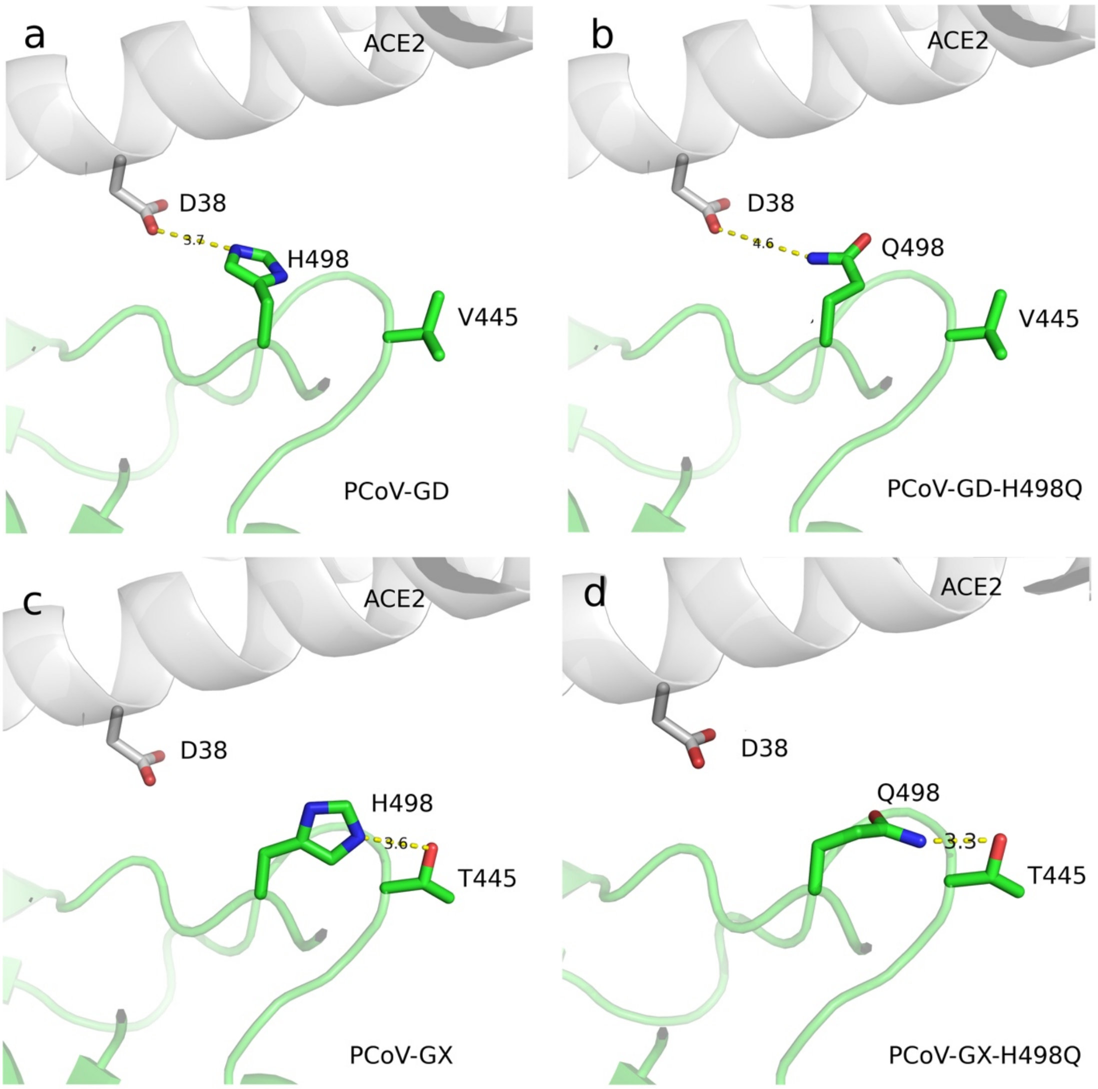


**Supplementary Fig. S2 The structure models of ACE2-RBD complexes for PCoV-GD, PCoV-GX and their mutants of H498Q. a** H498 of PCoV-GD connected to D38 of ACE2 with a salt bridge (Distance ~3.7Å). **b** Minor interaction between Q498 of PCoV-GD and D38 of ACE2 (Distance ~4.6Å). **c** H498 of PCoV-GD connected to T445 with a hydrogen bond (Distance ~3.6Å). **d** Q498 of PCoV-GD connected to T445 with a hydrogen bond (Distance ~3.3Å).
